# Feminine Deformity

**Published:** 1883-01

**Authors:** 


### FEMININE DEFORMITY.

The extraordinary perversion of taste and sense proved by the general opinion of what is desirable in female form may go far to prove that the principle of evolution is balanced by that of retrogression. The cave-men have left proofs of the possession of faculties not possessed by savage people of the present day, which may be taken as showing in the case of those who, incapable of improvement, die out before the march of civilization, that these latter are not going over the same ground of progress, but relapsing from a superior condition. The singular state of ideas respecting beauty of form in all modern civilized countries can hardly imply anything but retrogression in one of the senses at least. Judging of the opinion of the ancient Greeks as expressed in their sculpture, a modern, ideal, well-dressed young lady, probably by nature's intention as fine, or finer, than anything they ever saw, would be to them, could they revisit the earth, a subject of amazement! Tiny hands, white till they look bloodless, and pointed nails; feet with no more shape than a spoon; but, above all, a waist like a pipe, having scarcely any natural reference to the form above or below—in reality, hideous! The deeply-rooted preference for this deformity must surely be a mark of retrogression.

It is common for deluded mothers, looking at the grandly growing girl, to say, "The child is becoming a monster! she must be immediately put into stays." A little girl of twelve, being for the first time jammed into the abomination, complained that she could not breathe. The answer of her mother's French maid was, "Il faut souffrir pour être belle," and so commenced the deformity of the poor child's body and mind. There ought to be no such thing as a waist as now understood. In early youth flexible slimness is a natural characteristic, later it does not commonly exist, being replaced by a beauty of greater dignity; and when a small waist is formed by art it is at the expense of health and beauty. Every young lady who compresses her waist out of its natural shape and size should be made to understand that she does it at her peril, whether she feels the pressure or not, for from habit she may not be at all times conscious of it; she should know that she will pay a fearful price in loss of health and height and elasticity of movement, without which there can be no healthy pleasure and no real beauty. The test of beauty of form is the effect of the silhouette, and whether it will go well into sculpture; in fact, the effect of the lines bounding the shape. Compression on one place must produce corresponding expansion in another, except indeed in the disastrous crushing in of the ribs, which give way internally, sometimes entering the lungs. The ampler the form the less can good taste consent to compression. The sudden bulges and violent amplitudes which are the consequence of unnatural restrictions, are distressing alike to the sense of beauty and modesty—positively ugly—nature avenging herself! General amplitude is indeed far from ungracious, but, on the contrary, carries a dignity that is pleasant to look upon; but short, violent curves are eminently ugly.

